# Second-Class Care: How Immigration Law Transforms Clinical Practice in the Safety Net

**DOI:** 10.1177/00221465241254390

**Published:** 2024-07-27

**Authors:** Meredith Van Natta

**Affiliations:** Department of Sociology, University of California Merced, Merced, CA, USA

**Keywords:** cancer, immigrant health, medical legal violence, moral injury, public charge

## Abstract

This article examines how U.S. immigration law extends into the health care safety net, enacting medical legal violence that diminishes noncitizens’ health chances and transforms clinical practices. Drawing on interviews with health care workers in three U.S. states from 2015 to 2020, I ask how federal citizenship-based exclusions within an already stratified health care system shape the clinical trajectories of noncitizens in safety-net institutions. Focusing specifically on cancer care, I find that increasingly anti-immigrant federal policies often reshape clinical practices toward noncitizens with a complex, life-threatening condition as they approach a “specialty care cliff” by (1) creating time penalties that keep many noncitizens in a protracted state of injury and (2) deterring noncitizens from seeking care through threats of immigration enforcement. Through these processes, medical legal violence also creates the potential for moral injury among health care workers, who must adapt clinical practices in response to socio-legal boundaries of belonging.

Among many factors stratifying health care access in the United States, scholars increasingly emphasize the role of legal status in shaping noncitizens’ health (Castañeda et al. 2015; Davies, Basten, and Frattini 2010), especially in the context of primary care (e.g., Jimenez 2021; Kline 2019). Fewer studies focus on the intersection of citizenship and complex care, especially in the health care safety net, where publicly funded institutions and charitable organizations represent one of the few places where uninsurable noncitizens can seek such care (e.g., Melo 2017; Rodriguez 2015; Van Natta et al. 2019). Whereas safety-net institutions such as Community Health Centers, Federally Qualified Health Centers (FQHCs), county departments of health, and free clinics provide lower cost or free primary care to many low-income, uninsured noncitizens, specialty care is often far less accessible.

In this article, I examine how recent federal immigration and health policy changes shaped health care negotiations in distinct local safety-net contexts. Highlighting the relatively understudied area of specialty-care access among noncitizens, I focus on cancer diagnosis and treatment to illustrate how intersecting stratifications collide at one of the most difficult spaces for uninsured patients to navigate—what I call the “specialty care cliff.” Drawing primarily on interviews with 46 safety-net clinic and hospital workers in three U.S. states from 2015 to 2020, I ask how federal citizenship-based exclusions from an already stratified health care system shape the clinical trajectories of noncitizens with a complex, life-threatening condition as they approach this specialty care cliff. I find that increasingly anti-immigrant federal policies have accelerated medical legal violence in diverse local contexts, thereby codifying immigrants’ unequal belonging, altering clinical practices in health institutions, and contributing to moral injury among health care workers.

Existing scholarship has explored the social exclusion of immigrants whom the state constructs as “illegal” (e.g., De Genova 2013) and defined the “legal violence” of increasingly intertwining immigration and criminal law that permeates everyday life in immigrant communities of color (Menjívar 2021; Menjívar and Abrego 2012). Legal violence helps explain how superficially race-neutral laws criminalize many noncitizens who are racialized as non-White in the United States and generate symbolic and material harms associated with their supposed “illegality.” The harms of such criminalization include exploitative labor conditions, constant threat of deportation, and exclusion from many public programs. Scholars who have taken up legal violence in health care contexts emphasize how legal status often complicates clinical care for immigrants by stratifying health care opportunities and increasing concerns that seeking health care might trigger immigration enforcement (e.g., Gómez Cervantes and Menjívar 2020; Jimenez 2021).

Other scholars have used case studies to analyze these factors in relation to cancer diagnosis and treatment (Burke 2016; Gray et al. 2017; Jaramillo and Hui 2016; Jepson, Cox, and Peppercorn 2010; Olazagasti and Duma 2020), but they do not explicitly engage with the theory of legal violence. As Jimenez (2021:3) argues, however, viewing health care through the lens of legal violence enables scholars to analyze how “migrants experience illegality as they navigate medical bureaucracy.” Moreover, a small number of studies shows how laws that criminalize immigrants can also implicate health care institutions in these broader immigration enforcement forces (Jimenez 2021; Kline 2019; Van Natta 2023b). Such scholarship suggests the need for deeper attention to the high stakes of health care negotiations, where the imbrication of legal violence into clinical spaces as “medical legal violence” (Van Natta 2019) not only harms noncitizen patients but also compromises health care workers’ ability to uphold equitable care practices.

Specifically, medical legal violence enables medical-legal bureaucracies to potentially expand immigration enforcement surveillance through health institutions in ways that sometimes enroll health care workers as either agents or targets of that violence. Emerging scholarship has begun to explore the consequences of this spillover criminalization into health care institutions, emphasizing how medical legal violence not only harms excluded noncitizens but potentially co-opts health care workers into enforcing legal violence that is at odds with their professional duty to provide equitable care (Van Natta 2023a and 2023b). Yet when scholars apply the concept of medical legal violence, this latter point is seldom considered. Medical legal violence is not simply an extension of legal violence against noncitizens in clinical spaces; it also explains how immigration law unexpectedly reshapes clinical care itself.

The present article addresses this undertheorized aspect of medical legal violence by examining health care workers’ perspectives, emphasizing the necropolitical dynamics of clinical care at the specialty care cliff. Extending Foucault’s (1997:241) notions of biopower and biopolitics, which emphasize how the biopower to “make live and to let die” now accompanies the sovereign power to “take life or let live,” Mbembe (2003:21) advances the notion of necropolitics that stratifies populations to keep some people “alive but in a *state of injury*.” Medical legal violence extends necropolitics into clinical spaces, where the Hippocratic obligation to heal sometimes clashes with a broader immigration enforcement regime that lets die (Williams 2015). Previous scholarship has documented how this necropolitics harms excluded noncitizens in the United States and illustrated clinic workers’ resistance (Van Natta 2023a), but here I reveal the often underexamined corollary from health care workers’ vantage point: a system that lets die even despite their determined efforts to preserve life.

Within this context, cancer starkly illustrates the contradictions of care embedded within the highly politicized and stratified U.S. health care system. Although quantifying cancer rates among the foreign-born is difficult, previous research has estimated the proportion of foreign-born among those who died from cancer from 2005 to 2014 at about 9.3%, whereas the total foreign-born population ranged from about 12.1% to 13.0% during those years (Hallowell et al. 2019; U.S. Census Bureau 2021a, 2021b). Although the relative rate of cancer mortality was lower than that of the U.S.-born, it was higher for certain cancers—particularly those related to infection that could be prevented with improved health care access (Hallowell et al. 2019).

In this article, I suggest that understanding how medical legal violence operates through cancer care can help us better grasp how it also pervades the treatment differentials of more prevalent but diagnostically convoluted complex conditions at the specialty care cliff (e.g., diabetes, end-stage renal disease, cardiovascular illness). My findings reveal how federal policies that force many noncitizens toward danger at the specialty care cliff also often reshape clinical practices in distinct local contexts by (1) creating time penalties that keep many noncitizens in a protracted state of injury and (2) deterring noncitizens from seeking care through threats of surveillance and criminalization associated with immigration enforcement. These dynamics, in turn, compromise health care workers’ ability to adhere to disease-specific clinical practice guidelines and create the potential for moral injury.^1^

## Background

Even when uninsurable noncitizens can access primary care, they often face a specialty care cliff when their health issues require more complex services. Imagine ascending a hill, at the base of which are safety-net primary care facilities such as FQHCs and free clinics. Higher up the hill is secondary care, including specialty care and services like radiology or outpatient procedures. Finally, at the highest elevation is tertiary/quaternary care, which includes hospitalizations and long-term care. The path up this hill is dangerous, but under the right circumstances, there might be a safety net to catch those who fall. However, this safety net looks different depending on the person’s insurance coverage (or lack thereof). For U.S. citizens and long-term, lawful permanent residents, there might be a relatively broad and tightly woven net below in the form of employer-based insurance, state health exchange insurance, or Medicaid. Those who are functionally uninsurable, however, often have no net or must rely on a very small, very localized institution-specific net (“charity care”) to catch them. Many noncitizens who are uninsurable because of their federal ineligibility for comprehensive health insurance fall into this category.

Although Emergency Medicaid—a restricted form of the U.S. Medicaid public insurance program—is available to low-income patients irrespective of legal status, it does not cover ongoing care for chronic or otherwise long-term illnesses. Because federal law excludes many unauthorized and authorized noncitizens from all but Emergency Medicaid, they often must rely on local safety-net health institutions to manage their cancer care without the resources that are available to similarly situated U.S. citizens and lawful permanent residents. In this article, I demonstrate how these bureaucratic classifications supersede diagnostic categories to enact medical legal violence that codifies structural exclusions, stratifies clinical treatment, and leads to suboptimal care. I have presented patients’ accounts elsewhere (Van Natta 2019, 2023a, 2023b); here, I focus on clinic workers’ testimonies to demonstrate how medical legal violence transforms the clinical care they can provide to noncitizen patients with cancer and highlight the corresponding moral distress this entails.

### Federal Immigration Law, Medical Legal Violence, and Cancer

Federal laws determine which immigrants are qualified for programs such as Medicaid and Medicare, thus structuring what types of clinical care are available to which categories of patients. The 1996 Personal Responsibility and Work Opportunity Reconciliation Act (PRWORA) created the categories of “qualified alien” and “non-qualified alien” to restrict public benefits for undocumented immigrants, those with liminal statuses, and those with lawful status residing in the U.S. for less than five years. The 2010 Patient Protection and Affordable Care Act (ACA) later sustained these noncitizen exclusions and extended them to the newly formed health insurance exchanges (Kaiser Family Foundation 2023). Because states must work within these parameters and/or use state funding to pursue more inclusive programs, both immigration law and Medicaid administration increasingly constrain local safety nets’ ability to provide expansive care to unqualified immigrants (Varsanyi et al. 2012). Although some jurisdictions partially mitigate these federal exclusions through sanctuary policies and universal health coverage schemes (e.g., Houston et al. 2022; Marrow and Joseph 2015), the federal government retains substantial power to determine who gets high-level safety-net care.

Moreover, the 1986 Emergency Medical Treatment and Active Labor Act (EMTALA) requires that all hospitals receiving federal funds disregard immigration status or ability to pay when providing emergency medical treatment, but these laws only cover emergency- and pregnancy-related care. Thus, except for acute cancer symptoms that might require emergency medical intervention, much of the care involved in diagnosing and treating cancer falls outside the scope of Emergency Medicaid and EMTALA. Examining cancer navigation at the specialty care cliff therefore underscores how legal violence is codified in federal benefits policies in ways that institutionalize diminished health chances for immigrants—especially those with limited economic resources and/or those who are racialized as “people of color” in the United States (Menjívar 2021). Moreover, this legal violence is *medical* because it directly implicates health care workers and institutions through laws that appear unrelated to medical practice.

Health care workers who witness such structural inequities and experience clinical constraints on their basis may also experience “moral injury.” This condition, coined in the context of treating combat veterans who experienced sustained psychosocial impacts of “perpetrating, failing to prevent, or bearing witness to acts that transgress deeply held moral beliefs and expectations” (Litz et al. 2009:696), may also apply to health care workers under circumstances of sustained moral distress, wherein they know the right action to take but are situationally constrained from doing so (Jameton 1984). Ongoing experiences of moral distress can lead to moral injury, or emotional and psychological suffering associated with “being unable to provide high-quality care and healing in the context of health care” (Talbot and Dean 2018). Emerging scholarship has highlighted moral injury among health care workers in the context of COVID-19 (e.g., Akram 2021; Farrell and Hayward 2022; Shale 2020), but less is known about how more chronic structural constraints seemingly unrelated to health care practice—such as immigration law—may also generate moral injury in unique ways. This article’s focus on health care worker perspectives therefore emphasizes how medical legal violence not only creates a specific kind of “second-class care” for noncitizens at the specialty care cliff but also triggers the potential for moral injury among health care workers attempting to mitigate patient harms under intense structural constraints.

## Data and Methods

From 2015 to 2020, I investigated two interrelated questions: (1) How were uninsured/uninsurable immigrants navigating health care in distinct political contexts amid changing federal immigration and health policies, and (2) what were safety-net workers’ roles in facilitating that care? I conducted in-depth interviews (in English, Spanish, and Portuguese) and ethnographic observations in community clinics in three U.S. states and documented how Latinx immigrants and health care workers there addressed health challenges during a time of accelerating immigration enforcement. These methods allowed me to understand the on-the-ground experiences of both patients who sought care and clinic workers who navigated complex policies and bureaucracies to facilitate that care. Alongside interviews, ethnography revealed many of the mundane, otherwise invisible bureaucratic processes that subtly shape health care access in the safety net.

Although I initially aimed to explore opportunities for immigrant-inclusive social policies following executive orders supporting deferred immigration action and ongoing ACA implementation, heated debates surrounding the 2016 elections suggested significant federal policy changes in both spheres. The research focus therefore shifted to examine how policy uncertainty infused these negotiations, and here, I specifically examine the consequences of this uncertainty for specialty care in the safety net. By zeroing in on specialty care—the most inaccessible yet often highest stakes context of health care delivery—I illuminate underexamined aspects of care stratification. Beyond the physical harms this causes to impacted patients (Van Natta 2019, 2023a), I also convey its reverberations within clinical institutions and suggest the potential for moral injury to safety-net workers as an understudied yet fundamental consequence of medical legal violence.

The broader study also aimed to understand the influence of federal immigration and health policy changes on safety-net clinics in distinct local contexts. Previous research highlighted variation in local and state responses to the brightening of federal boundaries between citizens and noncitizens following the immigration and welfare reforms of the 1990s and ACA (e.g., Marrow and Joseph 2015; Park 2011; Varsanyi et al. 2012), suggesting that “nested” immigrant reception contexts (Golash-Boza and Valdez 2018) can either amplify or mitigate federal safety-net exclusions through local policies. Considering these implications, I pursued a multisited approach, selecting states according to their respective political governance—whether predominantly Democratic (“blue”), Republican (“red”), or mixed (“purple”) based on contemporaneous voting trends—and local immigrant politics (i.e., whether they directly collaborated with federal immigration agencies or resisted such partnerships; see Table 1.) Focusing on clinics within one county in each state, I conducted interviews with a total of 80 participants. Of these, 46 were clinic and affiliated community workers, and 34 were patients (33 from Latin America and 1 U.S.-born citizen in a mixed-status family).

**Table 1.** Description of Research Site Contexts.

Research Site Sample, Demographics, and Policy Context		
Blue State	Red State	Purple State
*n* = 18 clinic/community workers	*n* = 19 clinic/community workers	*n* = 9 clinic/community workers
Expanded Medicaid under the Affordable Care Act	Expanded Medicaid under the Affordable Care Act	Did not expand Medicaid under the Affordable Care Act
Provides state-funded Medicaid to certain undocumented children and adults	Does not provide state-funded Medicaid to any undocumented immigrants	Does not provide state-funded Medicaid to any undocumented immigrants
Provides Emergency Medicaid to income-eligible undocumented immigrants	Provides Emergency Medicaid to income-eligible undocumented immigrants	Provides Emergency Medicaid to income-eligible undocumented immigrants
Approximate % of Latinx residents in state = 40%	Approximate % of Latinx residents in state = 32%	Approximate % of Latinx residents in state = 10%
Approximate % of state population Latin American immigrants = 13%	Approximate % of state population Latin American immigrants = 8%	Approximate % of state population Latin American immigrants = 4%

*Source*: U.S. Census Bureau (2021c); National Immigration Law Center (2021).

Although I present patients’ perspectives elsewhere (e.g., Van Natta 2023a) and rely on the contextual information from these data for triangulation, here, I focus on conversations with health care workers in each state (n = 46). Many described challenges related to specialty care, and approximately 35% specifically addressed cancer. I draw from interviews with health care workers in primary care clinics and those who worked in specialty care settings, whom I often identified through snowball sampling. Thus, beyond the perspectives of primary care clinic workers that typify existing immigrant health research, I also include insights from those who coordinated cancer care in other settings, such as public hospitals and charity-care programs. At each clinic, many of the interactions that I observed and conversations that I had with health care workers involved instances of facilitating specialty care for federally unqualified patients with complex needs. Although these workers made the most of whatever services were available within their respective safety nets, several holes remained, making the specialty care cliff especially dangerous for excluded noncitizens.

The study received Institutional Review Board approval and a National Institues of Health Certificate of Confidentiality. Due to accelerating immigration enforcement activities, I refer to all participants and the states and clinics where they lived and worked using pseudonyms. I avoided asking for potentially identifying information—including race-ethnicity or immigration status—but such details often emerged indirectly during observations and/or interviews. Many patients described experiences of racial discrimination and exclusion—irrespective of and/or beyond legal status exclusions—that correspond to being racialized as non-White in the United States. Clinic workers, on the other hand, fell into two general groups: those who described themselves as members of the Latinx community they served and those who did not. The former often described dynamics of racial discrimination and structural racism similar to those that patients expressed, whereas the latter focused on inequities related to legal status and language access (for a more in-depth discussion of participants’ relative racialization experiences, see Van Natta 2023a).

I recruited patient participants from clinics during observations, and I recruited clinic workers through professional networks, snowball sampling, and during observations. Interview questions related to how participants first came to the clinic, how they navigated various health care institutions and bureaucracies (particularly when encountering obstacles), and how they understood these negotiations relative to specific immigration and health policies. Interviews were audio-recorded, transcribed in their original language, and coded using ATLAS.ti and constructivist grounded theory principles (Charmaz 2014). I also conducted situational mapping and social worlds and arenas mapping to grasp the relationships among social actors and among more abstract ideas, concepts, and discourses highlighting power dynamics embedded in structural and institutional arrangements (Clarke 2005:88). Intersecting, emerging codes related to processes such as “negotiating risk,” “navigating a cancer diagnosis,” and “facing a care cliff.”

In analyzing the subsample of interviews with 46 safety-net workers, I focused especially on participants’ responses regarding challenges that they have faced while facilitating care on behalf of immigrant patients and/or families. I asked participants to walk me through specific cases that came to mind as we spoke, and I also asked them about eligibility and health care coverage changes that they would make to bridge the gap between “how things work now and how you would like things to be.” More specific probing questions referred to relevant state and/or local policies, such as state Medicaid guidelines. Responses to these questions attuned me to analyze specialty care contexts more deeply, and through detailed coding and memo writing on this theme, cancer emerged as an analytical “ideal type” of specialty care that provided a clear snapshot of the complex issues safety-net workers described when discussing the unique stratification of high-level care for federally unqualified noncitizens. I therefore concentrate my analysis here on specific references to cancer, but the implications apply to similar challenges participants described related to end-stage renal disease, organ transplants, and so on.

Although participants only sometimes referred explicitly to a “cliff” to describe dangerous gaps in the safety net related to patients’ personal financial circumstances or the looming funding “cliff” that clinic workers feared during political battles over the ACA, situational and social worlds/arenas mapping enabled me to perceive this metaphorical cliff in broader terms related to the structural arrangements of safety-net care. Through repeatedly revisiting codes and reexamining them with reference to treatment of citizenship within health institutions and by bringing these data into conversation with the literature on legal violence in health care settings, I noted how cancer particularly exemplified this specialty care cliff in each state. I identified two mechanisms whereby medical legal violence at the federal level stratified noncitizens’ cancer care at the specialty care cliff: (1) through a necropolitical time penalty that delayed care and (2) through surveillance threats that pitted immigration enforcement concerns against the threat of grave illness. Safety-net workers also articulated how such challenges constrained their clinical care practices in distressing ways, suggesting the potential for moral injury.

## Results

### Medical Legal Violence and the Necropolitics of Time

In this section, I describe how medical legal violence and time collide at the specialty care cliff to keep unqualified noncitizens in a state of injury irrespective of local immigrant integration politics—albeit through somewhat distinct local mechanisms. Building on recent scholarship examining how U.S. immigration policies “let die” (De León 2015; Inda 2020; Kline 2019; Van Natta 2023a; Williams 2015), I argue that medical legal violence in the health care safety net creates a necropolitical time penalty that treats unqualified noncitizens as disposable. Rather than actively pushing them over the edge of the specialty care cliff, it leaves them hanging onto that edge. Meanwhile, safety-net health care workers whose job it is to facilitate care practices grounded in medical expertise and evidence instead race against the clock trying to pull those patients back from the edge.

In conversations with safety-net workers in the red state, for example, solid-tumor cancers emerged as a prime example of how the mismatch between demand and availability for charity care resulted in dangerous delays for unqualified noncitizens. In December 2017, nurse administrator Amanda described writing grants to fund colon cancer screening and diagnostic procedures, including colonoscopies and biopsies, for uninsured and functionally uninsurable patients like unqualified immigrants. Amanda recalled a patient who was screened through a grant-funded colonoscopy program. “She had never had any screening,” Amanda explained, but once the colon biopsy indicated cancer, Amanda’s team “performed a miracle” to coordinate treatment by patching together donated care from several sources. Amanda’s comments emphasized that achieving such complex clinical care for federally unqualified immigrants represented the exception rather than the norm. Specifically, it required health care workers to summon miracles at the edge of the specialty care cliff—particularly via nonstate mechanisms—while patients struggled to hold on. These laborious workarounds created dangerous delays for patients when time was most of the essence.

Red-state nurse Marie, whom I met at a free clinic in 2018, explained that the fractured health care that was sporadically available to many noncitizen patients reflected a distinct tier of clinical care, even within an already stratified and fragmented health care system. In contrast to U.S. citizens and lawful residents with Medicaid and/or other forms of insurance who typically received reminders regarding relevant screening guidelines, Marie described that “when it comes to immigration health care, it’s like all of that goes out the wayside”:

[So] we have a lot of late cancers diagnosed, more advanced disease diagnosed. Of course, it’s harder to treat. Of course, their long-term outcomes and quality of life are reduced because of late diagnoses. That’s sort of the huge thing that I battle is late cancer diagnoses. . . . Then, they end up needing surgery and stuff, and finding that more advanced specialty care is an even bigger mountain to climb in this [undocumented] population.

Marie recalled patient Mireya, whom she had been treating in the wound-care clinic. Mireya had a breast mass and managed to get a mammogram and biopsy to diagnose cancer, but she was unable to arrange treatment. While Mireya awaited charity care at the county hospital, her tumor grew. “She ended up having one of the most terrible wound complications post-mastectomy,” Marie recalled:

The cancer was so expansive and really, kind of eroded in. It was a complicated surgery, and so as a result she had a very difficult wound. We did end up closing it, but she has really terrible scarring, and that would never happen if she had insurance. She would have been treated early, she would have had access to a plastic surgeon, she would have been reconstructed.

Even though Marie described how being uninsured shunted Mireya to a lower tier of care and a diminished health outcome, Mireya could more accurately be described as *uninsurable* than uninsured. Unlike similarly situated U.S. citizens and long-term lawful residents, Mireya’s legal status and residence in a necropolitical regime, where both federal and local immigrant health policies barred her from subsidized care, forced her to rely on institution-specific charity care. This, in turn, involved a time penalty that allowed her tumor to expand and derailed her ability to heal in the ways that someone with a qualifying legal status might.

Marie added that in her experience, noncitizens with colon, breast, and ovarian cancer frequently faced diagnoses in the most advanced stages of their cancer. She remembered 54-year-old patient Francisca, who died after being diagnosed with late-stage ovarian cancer. It took a long time for Marie to coordinate chemotherapy for Francisca, and it was delayed well beyond the clinical practice guidelines that providers like Marie rely on when crafting treatment plans. Marie again drew the comparison between insured U.S. citizens and nonqualified immigrants, saying, “Whereas you and I would start [treatment] like a week later, her we had to get funding, and money, and convince people to do it at a discount.” As a clinician, Marie stressed that these kinds of delays should not happen. “The delays begin with there’s no way these people can access health insurance,” she reiterated. “You know, you have to be a full citizen to buy in. That’s one piece. County has discounted programs; those have huge wait lists.” For unqualified noncitizens who were ineligible for Medicaid and most other forms of insurance because of legal violence, cancer care was therefore stratified both on the front end during the diagnostic process and the back end, seeking treatment for which they had neither the legal status—nor the time or money—to wait.

Like Marie, free-clinic director Dr. Francis recalled several patients who faced delayed care because their legal status made them functionally uninsurable. She described one patient with a “humongous thyroid mass, which I’m sure is going to be cancer.” Dr. Francis was trying to coordinate a biopsy, but this involved costly specialty care at an already beleaguered county safety-net hospital. “Maybe she’ll be able to come up with whatever amount of money that they quote her for the biopsy,” Dr. Francis continued, “but the big problem is if it shows cancer, then what do we do?” She currently faced this situation with another patient, for whom she had managed to arrange a kidney biopsy through the county safety net. “Now we know he has cancer,” she said, “but now he has to somehow try to afford a surgery to remove his kidney and whatever else and it’s just . . . ,” Dr. Francis trailed off bleakly. Her unfinished sentence reflected the futile sense of uncertainty providers perceived while trying to facilitate care for patients with cancer at the specialty care cliff, where they could do little to ameliorate this state of injury and the dangerous waiting game that accompanied it.

The reflections of purple-state health care workers, meanwhile, suggested how medical legal violence can create another type of necropolitical time penalty unique to unqualified noncitizens. In May 2020, oncology social worker Nadine described bone marrow transplants for leukemia and lymphoma as particularly challenging. Although Medicaid covers bone marrow transplants for leukemia and lymphoma, Emergency Medicaid does not. Nadine walked me through the distinction between the two:

[Emergency Medicaid] only covers you for certain numbers of days that you are required to be hospitalized through the emergency room for certain types of things. Let’s say all of sudden . . . you’ve developed leukemia. You go to the emergency room because if you don’t, you’re going to die, and you get hospitalized for a month, and in that first month you start chemo and all these other things. Well, it might be only the first three days of that hospitalization that would be covered by Emergency Medicaid. . . . Then once somebody is stabilized, even though they’re still in the hospital, and even though they’re going to die if they leave the hospital, it’s not covered.

Nadine explained that for many other conditions, this was when the institution’s financial assistance program came into play. Because these transplants cost hundreds of thousands of dollars without insurance, however, financial assistance programs would not cover that component of a patient’s care. Although Nadine could often arrange sufficient charity coverage for chemotherapy that might enable remission for patients with blood cancer, curing them required a bone marrow transplant. The distinction between remission and cure highlighted how “medically necessary” treatments often depended more on a patient’s bureaucratic classification than clinical guidelines. As Nadine explained:

There isn’t really a “why.” It’s basically determined by the hospital administration that we’re not going to do this as “medically necessary,” which is interesting and has all kinds of ethical implications. We have lots of undocumented immigrants who are on these second-class citizen, if you will, or not as good chemotherapy regimens for these blood cancers because they can *never* get a bone marrow transplant because they can’t pay for it.

Nadine explained that awareness of these “second-class citizen” treatments, which evoked a Kafkaesque necropolitical time loop that extended unqualified noncitizens’ state of injury indefinitely, created what Nadine described as “very moral distress” for her and her oncology colleagues. When I asked Nadine to describe any cases when this bureaucratic definition of what was “medically necessary” impacted the care she could coordinate, however, she pushed back on the way I phrased the question. “It’s more than bureaucratic,” she replied emphatically:

It’s a financial definition. It’s where finances meet. It’s political, right? I mean, finance is like it’s medically necessary, but only if you have the money to pay for it or the insurance. That’s when it’s medically necessary, but if you don’t then it’s not medically necessary. We’ll treat you with this other thing that’s not as good. That’s essentially what it is.

Nadine’s remarks underscore two important points. The first is how medical legal violence compounded existing economic stratifications to further disadvantage unqualified noncitizens with cancer. The second relates to how this arrangement constrained the clinical care that medical workers like Nadine could coordinate. Even though her clinic offered relatively robust safety-net care to qualified low-income, uninsured residents throughout the state, federal citizenship-based Medicaid restrictions limited what she could realistically achieve for federally unqualified noncitizens. This situation, in turn, caused Nadine “moral distress” when she knew what course of care was clinically recommended but was unable to coordinate it because of legal status stratification.

Like Nadine, purple-state oncologist Dr. Lopez described similar situations that illustrated how medical legal violence could result in the aforementioned “letting die” by compounding the existing hazards of potentially fatal conditions. “When [the cancers] progress or don’t respond to standard treatment anymore,” he explained, “the patients can’t qualify for more treatments anymore, just because of their lack of insurance. If they had [Medicaid] and had appropriate documentation, they would be able to qualify for clinical trials.” Echoing Nadine’s comments about the refusal of Emergency Medicaid and hospital-specific financial assistance programs to cover the clinically indicated treatment for blood cancers (i.e., bone marrow transplant), Dr. Lopez recalled 26-year-old undocumented patient Ilaria who arrived in the United States as a child and was recently diagnosed with Hodgkin’s lymphoma. Ilaria was hospitalized upon diagnosis and responded well to inpatient treatment. Soon after discharge, however, she appeared in the emergency department again requiring another course of treatment. Ilaria was now caught in a cycle of emergency, admission, treatment, discharge, and recurring cancer that demanded exceptional efforts from the oncology social workers to facilitate given her functional uninsurability. “So, for now we’re keeping her on a drug that’s calming her illness,” he added, “but we know it’s not curative, and it’s been like this for a year already.” When I asked Dr. Lopez what the long-term plan was for Ilaria, he replied that the prognosis was grim. “The most probable thing, knowing this illness . . . is that she will die from it.”

Medical legal violence thus generated various time penalties for unqualified noncitizens with cancer, and witnessing this stratification with few institutional remedies weighed heavily on health care workers whose primary role was to heal. Even where state and county immigrant health funding was more robust, the power of federal exclusions often overwhelmed providers. For example, Dr. Carrera, chief medical officer of the blue-state clinic that I observed, emphasized the limitations that federal immigration and health care laws placed on a relatively immigrant-inclusive safety net. Dr. Carrera reflected on how difficult it was “for anybody in health care to wanna be in health care when there’s two tiers of health care that are being practiced at the same time.” Like Marie and Nadine, who explicitly described distinct tiers of care at the specialty care cliff based on citizenship status, Dr. Carrera’s remarks underscored the compounded stratification of health care that he witnessed. He added that the emotional impact of this medical legal violence spilled beyond patients onto safety-net clinic workers as well:

If [patients] need to go to a tertiary care center or a hospital or something else like that, that’s really difficult for a human being to sleep at night. . . . Or if I can’t get somebody who’s got multiple myeloma [a cancer of blood plasma cells] into an oncologist’s office for treatment, and it’s taking weeks and weeks and weeks, it stresses the front office because they get calls from family members who are crying. Or nurses who get calls trying to get somebody [into] charity care. . . . But it just delays care for weeks, sometimes a month or so. It’s not good for anybody when you have this delay because of not being able to put somebody on an insurance plan.

Dr. Carrera’s comments highlight how federal laws pervade local safety-net institutions through medical legal violence and constrain health care workers’ efforts while suspending unqualified noncitizens in a dangerous biological limbo. Even within a relatively immigrant-inclusive safety net, the additional time and effort that clinic workers must expend to pull patients back from the edge of the specialty care cliff contradicts clinical practice guidelines that demand timely, continuous treatment. Clinic workers therefore struggled to navigate delays and vicious cycles that, echoing Mbembe’s (2003:21) words, kept patients “alive but in a *state of injury*.” In doing so, these health care workers sometimes experienced corresponding symptoms of moral injury when they knew the optimal course of cancer treatment but were unable to coordinate it due to circumstances beyond their control. Beyond the everyday resource challenges typical of safety-net care coordination, however, in this case, it was restrictive immigration laws that constrained care at the specialty care cliff by explicitly excluding “unqualified” noncitizens.

### Balancing Fears: Immigration Enforcement versus Cancer Care

Beyond harmful time penalties, the necropolitical dynamics that medical legal violence sustains—and the moral injury it generates—also rely on the perpetual threat of immigration enforcement to disproportionately discipline noncitizens. Indeed, when Dr. Carrera expressed the emotional strain that providers experienced through working against the legal status-based time penalties that he and other participants described, he also explicitly emphasized how anti-immigrant laws exacerbated patients’ fears of becoming visible to immigration enforcement agencies through their health care utilization. This further undermined the clinical practices of health care workers trying to facilitate care during a time of intensifying federal anti-immigrant politics.

I spoke with Dr. Carrera in summer 2018—when the Trump administration’s separation of families at the U.S.–Mexico border dominated global news cycles. Dr. Carrera directly connected escalating federal immigration enforcement with the ACA and broader insurance exclusions that had existed in the United States for decades. He explained that although the ACA had positively transformed health care, including the expansion of Medicaid and health insurance exchanges to previously ineligible citizens and lawful permanent residents, it also left out many immigrants in his community. As the Trump administration intensified anti-immigrant health, welfare, and immigration enforcement policies, Dr. Carrera became frustrated that many immigrant patients in the community were “hiding” and more reluctant than ever to go to a community clinic. “Because they’re scared and not willing to fill out [state-funded Medicaid documents],” he explained, “or fill out paperwork or talk to somebody about why it is that they have to fill out all this information to be able to get into [charity care].”

Dr. Carrera’s comment referred to the fact that unlike Emergency Medicaid, accessing state-funded specialty care for cancer treatment required unqualified noncitizens to disclose their legal status to Medicaid agencies. Although blue-state eligibility criteria permitted this, its contradiction to federal eligibility criteria provoked substantial anxiety among patients. This was especially true after the 2016 election, when many expressed concerns that having sought state-funded care through a federally administered agency would put them on immigration enforcement’s radar (Van Natta 2019). Although clinic workers were often able to persuade patients to complete such documentation requirements before the 2016 election, Dr. Carrera emphasized how accelerating medical legal violence stoked new anxieties around this level of visibility that then deterred eligible patients from seeking care. Thus, despite a relatively robust safety net at the edge of the specialty care cliff in the blue state, many noncitizens began to view that net as a potential tool to entrap them rather than protect them. These dynamics underscore the pervasive power of federal lawmaking to shape local immigrant policies—even in jurisdictions with immigrant-inclusive politics—in surprising ways.

Although Dr. Carrera emphasized the extreme measures of the Trump administration to highlight patients’ acute enforcement concerns, earlier conversations with his colleagues suggested that this deterrence was long in the making. For example, during an interview in October 2015, certified enrollment counselor Isabel identified the state’s breast and cervical cancer programs as one example of how a sense of enhanced surveillance around the perceived threat of immigration enforcement could undermine state-funded eligibility. She explained that low-income, federally unqualified noncitizens with a cervical or breast cancer diagnosis could receive several months of state-funded Medicaid to undergo treatment, but they needed substantial guidance to ensure they understood what (if any) immigration-related consequences might arise. Isabel stressed that seeing the words “immigration status” on forms often discouraged patients from applying for vital care. She recalled multiple cases in which eligible patients with a cancer diagnosis refused to apply for state-funded care because they feared that doing so would lead to deportation. Isabel expressed frustration that patients with cancer had to worry about this when what they most needed was “a moment to focus on themselves and on their recuperation, if there is to be any recuperation.”

Isabel’s comments suggested that even when patients were eligible for state-funded care through the exceptional circumstance of their cancer diagnosis, clinic and oncology teams often had a difficult time persuading them to pursue this opportunity for life-saving treatment. Oncology social worker Kelly, who collaborated closely with Isabel’s clinic and Medicaid administrators, found it “really challenging” to assuage patients’ concerns during these conversations:

They weigh the benefit of declaring [their status in the application] versus the risks of immigration services. The fear of being deported, and the immigration [agencies] finding out about them is a great concern, and I think that prevents a lot of people from [applying]. As much as I try to explain it to them, and I work with the [Medicaid] representatives of the county . . . it’s still very hard for them to declare [their undocumented status].

Blue-state referrals specialist Antonio, who worked with Dr. Carrera and Isabel, echoed these impressions in an interview in November 2015. Antonio reflected on patients’ security concerns from his own vantage point as a Latin American immigrant who had struggled to become insured. From his perspective, the “main obstacle” to patients with serious medical conditions like cancer was “to overcome the fear that the patient [has] of being known as an illegal immigrant,” he explained. “Because first the patient needs to accept being—in [my language] we call it . . . like [being under a fine mesh],” Antonio remarked. “Meaning you cannot hide yourself anymore, and that’s huge, right?” In Antonio’s experience, noncitizens’ perception of being constructed and treated as “illegal” by federal immigration policies undermined state and local efforts to bolster the safety net for all residents regardless of citizenship classification.

Whereas the blue state had the most expansive immigrant-inclusion criteria, the sparser safety nets in the red and purple states relied more on institution-specific (rather than state-funded) “charity care” or financial assistance programs. Yet—further emphasizing the pervasiveness of federal lawmaking in local institutions—participants in these states also reported increasing requirements for undocumented noncitizens to disclose their status to access institution-specific financial assistance programs. Clinic workers in both states reported recent changes to “charity care” applications in the safety net that required patients to apply for full-scope Medicaid—even when they knew that they would be denied based on their legal status and even when doing so risked making undocumented individuals visible to federal agencies—to provide the denial letter as proof of eligibility for charity care.

Dr. Francis recollected red-state patients reaching the edge of the specialty care cliff after delaying care on the presumption that their legal status would prevent them from getting any type of care. She explained that undocumented immigrants often internalized anti-immigrant messaging in local and national media and its accompanying confusion and misinformation about benefits eligibility. Her reflections suggested that when patients perceived themselves and/or their engagement with safety-net institutions as “illegal,” they often forewent care until they were, in her words, “at death’s door.” Dr. Francis emphasized that this sometimes had devastating consequences:

There are a lot of people who we have to send directly to the emergency room because, at that point in time, there’s nothing that we can do to help as well. And I have one lady who ended up having liver cancer, and I was able to get her seen by [a private hospital’s] charity clinic, but by that point in time she was so far advanced that there was nothing that they could do for her. So essentially, she had an appointment with the surgeon who said, “You’re unresectable, there’s nothing we can do.”

Dr. Francis’s remarks highlight how medical legal violence, codified in laws that exclude many noncitizens from health and well-being services, is both material and symbolic, differentially shaping the health chances of unqualified noncitizens who become stranded at the specialty care cliff. “[It’s] still not like it should be, like we were taught in medical school,” Dr. Francis lamented later in our conversation. She emphasized that such situations defied “the way that we were all taught that you practice medicine, not that you wait until they acutely are ill, and you have to do it in an emergency setting. . . . [It’s] not the best practice.” Such reflections highlight the indirect yet consequential emotional strain that such necropolitical conditions place on health care workers who witness these delays in care as a function of a political reality over which they have little direct control.

Likewise, Nadine described how concerns over the “public charge” rule made many purple-state patients reluctant to seek health care and well-being support on the rare occasions it was available to them. This rule, which penalizes some noncitizens seeking an entry visa or permanent residency based on their use of certain public benefits and which the Trump administration expanded in 2019 (U.S. Department of Homeland Security 2019), did not apply to most of the federally “unqualified” noncitizens who Nadine’s hospital served. Yet despite this and despite the high stakes of life-and-death care in the oncology clinic, Nadine struggled to dispel patients’ anxieties and optimize the care for which they were eligible:

There’s been all this stuff about the public charge rule and the current [Trump] administration, so people are afraid to use benefits. . . . And we’ve had people afraid to bring their children in for treatment. If they have children with cancer, who are [purple state] residents. They’re afraid that [if] their children get help or get Medicaid, that they will someday make it impossible for them to get legal residency and stuff like that.

Nadine’s remarks emphasized the spillover effects of medical legal violence, wherein the perception of immigration enforcement surveillance within health care settings extended beyond an individual with cancer and implicated entire families—especially those with mixed immigration statuses—and constrained clinical care opportunities even for those who might be eligible for more expansive care.

Nadine’s colleague, Dr. Lopez, also perceived increasing reluctance toward institutional visibility, which he attributed to a sense of enhanced immigration enforcement surveillance under the Trump administration. As he remarked:

When the administration changed [from Obama to Trump], people who might have already had economic difficulties and [were] trying to acquire citizenship or have a better position in this country, lost their hope when the government changed. . . . I think that was almost unanimous among people I was [treating]. Many of them [began] asking you, “Isn’t there another treatment that doesn’t require any paperwork, or that I can find or pay for on my own?”

Such remarks underscore that beyond the time penalties that participants witnessed among unqualified patients with cancer, the necropolitical possibility of “letting die” also operated through the threat of inscribing patients into medical bureaucracies that frequently seemed antagonistic rather than curative.

Emphasizing again the pervasive reach of federal lawmaking into divergent political contexts, the perception that specialty care required increasingly onerous disclosures from noncitizen patients permeated safety-net institutions across all three study sites. Much like the “prevention through deterrence” immigration enforcement strategies of the mid-1990s created a necropolitical state of exception through border militarization (e.g., De León 2015; Williams 2015), accelerating medical legal violence exacerbated enforcement fears and deterred many noncitizens and mixed-status families from seeking life-saving care. Although scholars have documented this “chilling effect” among immigrant communities (e.g., Haley et al. 2020), safety-net health care workers’ perspectives illuminate the broader structural and institutional arrangements that generate and sustain dangerous exclusions for “unqualified” noncitizens. Moreover, these perspectives shed light on both the scope of the problem and the obstacles that such bureaucratic antagonism creates for safety-net personnel who must find clinical workarounds to accommodate political uncertainty. This especially complicates specialty care in the safety net, where health care workers already face intense resource constraints, and compounds these difficulties through an unlikely factor: federal immigration law.

## Discussion

By examining cancer navigation at the specialty care cliff, I have argued that medical legal violence at the federal level transforms clinical care practices even in diverse local contexts. The testimonies of safety-net health care workers demonstrate how legal violence becomes medical legal violence when it directly implicates health care workers and institutions to create treatment differentials that stratify health by legal status. Building on previous scholarship examining legal violence in (typically primary care) health care contexts, I focus on cancer to further explain how medical legal violence sustains a necropolitical arrangement that disproportionately harms Latinx noncitizens. Yet whereas others have examined the necropolitical immigration regime in more punitive contexts of enforcement and detention (e.g., Inda 2020; Williams 2015), I examine its extension into clinical spaces that are ostensibly meant to heal rather than punish. By highlighting the perspective of health care workers, I illuminate previously unexamined aspects of stratification in specialty care settings while drawing attention to how medical legal violence also spills over onto clinic workers in unique ways—including the potential for moral injury.

Medical legal violence helps explain how and why stratified pathways to specialty care diverge at multiple points along the way from primary-care access to treatment options depending on how legal and biological circumstances intersect. This violence operates through two pathways to transform clinical care: (1) through various time penalties that force and/or sustain patients in states of injury and (2) through surveillance and enforcement fears that deter patients from seeking care. In these ways, the codified exclusion of unqualified noncitizens from federally subsidized care, alongside thinly stretched state safety nets that likewise exclude these individuals from many avenues to care, results in embodied harms. It also compounds challenges for safety-net health care workers who must further adapt their clinical practices to negotiate these socio-legal boundaries of belonging.

Beyond cancer, medical legal violence also helps explain a variety of citizenship-related health inequities in the United States—particularly in the wake of COVID-19. For example, this framework provides crucial insights into its disproportionate impact on Latinx immigrant communities in the United States (Page and Flores-Miller 2021). Reflecting Mbembe’s (2003:27) observation that the state of exception serves to “define who matters and who does not, who is *disposable* and who is not,” rhetoric praising “essential workers” during the pandemic has obscured the reality that many workers deemed “essential” are also classified as “unqualified” for vital health and welfare benefits (Yearby and Mohapatra 2020). Similarly, just as the pandemic has made many aspects of medical legal violence visible in real time, it has intensified the likelihood of moral injury among another sector of so-called “essential workers”: health care workers who have had to change their care practices in response to rationed resources, medical uncertainties, and political turmoil (Akram 2021; Farrell and Hayward 2022).

Yet, although the pandemic cast new light onto long-standing health care inequities and placed much of the ethical burden on health care workers to navigate, it is somewhat more challenging to assess the potential for moral injury via inequitable social policy—in this case, federal immigration law. On the surface, clinical practice and immigration law appear generally unrelated, making it easy to misrecognize the material consequences of their intersection. Yet this is precisely how medical legal violence operates, through the almost imperceptible incursion of socio-legal categories into clinical spaces. By examining health care workers’ perspectives on this incursion and tracing the moral distress that accompanies its impact on clinical care, we can better understand how more abstract social policymaking shapes clinical care in vital moments. When analyzed through the conceptual lens of medical legal violence, these perspectives reveal how U.S. immigration laws codify necropolitical citizenship stratifications through time penalties and enforcement threats, thus transforming clinical care and further stratifying care pathways in an already inequitable health care system.

Moreover, the examples detailed here shed light on the spillover consequences of this necropolitical regime for health care workers, who sometimes experience moral injury when confronted with unanticipated constraints to clinical care. Although I have focused here on how federal anti-immigrant politics stratify specialty care for noncitizens through medical legal violence, the implications of a necropolitical health care regime that renders certain groups uniquely vulnerable to injury extends beyond immigrant communities. Laws that deny vital gender-affirming and reproductive health care throughout the United States, for example, underscore Mbembe’s (2003) insights on constructed disposability. Much as immigrant-exclusionary laws create a type of “second-class care” for many noncitizens seeking cancer diagnosis and treatment, recent state laws that defy clinical practice guidelines related to gender-affirming and reproductive care (e.g., Keith 2023; Wright Clayton, Embí, and Malin 2023) compromise patients’ safety while intensifying the potential for moral injury among health care workers who cannot provide the care they know is appropriate and necessary.

Taken together, these insights suggest parallel paths toward the specialty care cliff for those whom the state constructs as disposable. Future research should explore these necropolitical formations in various health care spaces to better understand their possible consequences for clinical care. By examining these dynamics through the lens of medical legal violence, we can better assess not only the embodied consequences of discriminatory social policy on targeted individuals but also the spillover effects on those whose professional practices are constrained by such policies.

## Notes

1. Clinical practice guidelines are recommendations, based on current medical evidence and expertise, that guide health care practitioners in diagnosing and treating medical conditions. They are not binding requirements, but they do articulate certain clinical standards from which deviations “must be justified” (National Library of Medicine 2020; see also Institute of Medicine 2011; Reames et al. 2013).
